# 

**Published:** 1880-05

**Authors:** 


### Table of Contents.

### ORIGINAL COMMUNICATIONS.

A Case of Puerperal Eclampsia, with Remarks. By Joseph Eve Allen, M.D., Augusta, Ga ........ 65
Account of a Peculiar Form of Malarial Pneumonia, with a Suggestive Plan of Treatment. By W. D. Hoyt, M.D., Rome, Georgia ........ 73
The New Anæsthetic. By W. E. Riggs, D.D.S., Atlanta, Ga........ 76

### REPORTS OF SOCIETIES.

Transactions of the Obstetrical Society of Cincinnati........ 79
Vanderburgh County Medical Society ........ 91
St. Louis Medical Society........ 95
New York Academy of Medicine........ 104

### SELECTIONS.

Chorea and Whooping Cough........ 114

### EDITORIAL.

Medical Association of Georgia........ 120
Meteorological Report for April........ 122

### BIBLIOGRAPHICAL.

Wood's Library of Standard Medical Authors for 1880........ 123
The Venerial Diseases, including Stricture of the Male Urethra........ 123
A Treatise on Foreign Bodies in Surgical Practice........ 123
A Handbook of Physical Diagnosis........ 123
Post Mortem Examinations, with Special Reference to Medico-Legal Practice........ 124
Sea-Air and Sea-Bathing........ 124
Our Homes........ 124

### EXCERPTA.

Nostrums........ 124
The Physiological Action and Therapeutic Value of Sclerotic Acid.. 125
On a Visual Phenomenon and its Explanation........ 127
Placenta Prævia - Version—Craniotomy—Death of Mother........ 128

